# Prospective validation of dermoscopy-based open-source artificial intelligence for melanoma diagnosis (PROVE-AI study)

**DOI:** 10.1038/s41746-023-00872-1

**Published:** 2023-07-12

**Authors:** Michael A. Marchetti, Emily A. Cowen, Nicholas R. Kurtansky, Jochen Weber, Megan Dauscher, Jennifer DeFazio, Liang Deng, Stephen W. Dusza, Helen Haliasos, Allan C. Halpern, Sharif Hosein, Zaeem H. Nazir, Ashfaq A. Marghoob, Elizabeth A. Quigley, Trina Salvador, Veronica M. Rotemberg

**Affiliations:** Dermatology Service, Department of Medicine, Memorial Sloan Kettering Cancer Center, New York, NY USA

**Keywords:** Preclinical research, Physical examination

## Abstract

The use of artificial intelligence (AI) has the potential to improve the assessment of lesions suspicious of melanoma, but few clinical studies have been conducted. We validated the accuracy of an open-source, non-commercial AI algorithm for melanoma diagnosis and assessed its potential impact on dermatologist decision-making. We conducted a prospective, observational clinical study to assess the diagnostic accuracy of the AI algorithm (ADAE) in predicting melanoma from dermoscopy skin lesion images. The primary aim was to assess the reliability of ADAE’s sensitivity at a predefined threshold of 95%. Patients who had consented for a skin biopsy to exclude melanoma were eligible. Dermatologists also estimated the probability of melanoma and indicated management choices before and after real-time exposure to ADAE scores. All lesions underwent biopsy. Four hundred thirty-five participants were enrolled and contributed 603 lesions (95 melanomas). Participants had a mean age of 59 years, 54% were female, and 96% were White individuals. At the predetermined 95% sensitivity threshold, ADAE had a sensitivity of 96.8% (95% CI: 91.1–98.9%) and specificity of 37.4% (95% CI: 33.3–41.7%). The dermatologists’ ability to assess melanoma risk significantly improved after ADAE exposure (AUC 0.7798 vs. 0.8161, *p* = 0.042). Post-ADAE dermatologist decisions also had equivalent or higher net benefit compared to biopsying all lesions. We validated the accuracy of an open-source melanoma AI algorithm and showed its theoretical potential for improving dermatology experts’ ability to evaluate lesions suspicious of melanoma. Larger randomized trials are needed to fully evaluate the potential of adopting this AI algorithm into clinical workflows.

## Introduction

The clinical diagnosis of melanoma is challenging, resulting in dozens of unnecessary skin biopsies performed for every melanoma identified^1^. Technological approaches to improve non-invasive evaluation of skin lesions suspicious for melanoma have included multispectral imaging, reflectance confocal microscopy (RCM), electrical impedance spectroscopy, and adhesive skin patch testing^2,3^. Despite their promise, none of these tools have gained widespread clinical adoption. The application of artificial intelligence (AI) to images of individual skin lesions has significant advantages since it can be performed with existing images captured during routine clinical care without significantly added expense or time. Image-based AI of individual skin lesions has been shown to both exceed and improve expert dermatologist performance in artificial experimental settings^4^. However, concerns have been raised about bias due to nonpublic datasets, proprietary algorithms, and lack of external validation via prospective clinical studies^5,6^.

Through the International Skin Imaging Collaboration (ISIC), we hosted five annual challenges to support the development of AI for melanoma detection^7–11^. The top-ranking AI algorithm of the 2020 challenge, “All Data Are Ext” (hereafter ADAE), was trained exclusively on public dermoscopy images. As this algorithm is open-source, non-commercial, and amenable to rapid adoption into existing workflows, it has significant potential to improve the selection of lesions that undergo biopsy to rule out melanoma. Here, we aimed to prospectively validate the accuracy of ADAE for melanoma diagnosis and to assess its potential impact on dermatologist clinical decision making.

## Results

### Participant characteristics

437 patients consented to the prospective study but two were excluded due to ineligibility, leaving 435 participants with 603 lesions for analysis. Participants had a mean age of 59 (IQR: 50–71) years, 54% were female, and 96% were White individuals (Table 1).

**Table 1:** Characteristics of prospectively accrued study participants.

Characteristic	*N* = 435	Col %
Age [mean (IQR)]	59	(50, 71)
Sex		
Female	235	54
Male	200	46
Race		
Asian	4	1
White	416	96
Other	2	0
Unknown	13	3
Ethnicity		
Hispanic	6	1
Non-Hispanic	419	96
Unknown	10	2
Study dermatologist		
Derm1	251	58
Derm2	81	19
Derm3	47	11
Derm4	23	5
Derm5	18	4
Derm6–Derm11	15	3
Fitzpatrick skin phototype		
I	38	9
II	240	55
III	139	32
IV	18	4
Nevus phenotype		
Florid	49	11
Moderate	87	20
Mild	187	43
No/few	112	26
Personal history of melanoma	209	48
Family history of melanoma	157	36
Number of enrolled lesions		
1	321	74
2	79	18
3	20	5
4+	15	3

Nevus phenotype was the dermatologist’s subjective assessment of the number of total moles and presence of atypical moles.

### Lesion characteristics

Ninety-five melanomas and 508 non-melanomas underwent biopsy and were enrolled in the study. Melanomas were most frequently in-situ (*n* = 49, 52%). Of 46 invasive melanomas, the median thickness was 0.56 mm (range: 0.2–7.3 mm). Two were ≥1 mm in thickness. Non-melanomas included 312 nevi, 45 lentigines, 28 atypical melanocytic proliferations, 23 seborrheic keratoses, and 22 keratinocyte carcinomas (Supplementary Table 2).

### Dermatologist characteristics

Participants were recruited by 11 dermatologists; 5 recruited ≥20 lesions. The 5 dominant dermatologists enrolled 97% of study lesions (range: 22–389 lesions), 94% of melanomas (range: 0–29), and 98% of non-melanomas (range: 11–366). The 5 dominant dermatologists had a mean number of years of post-residency clinical experience of 16 (range: 3–33 years), and all had a primary clinical focus on skin cancer. Their number needed to biopsy (NNB) ranged from 1.9 to 16.9 and the number of melanomas diagnosed per 100 clinic visits ranged from 1.5 to 1.8.

### Accuracy of the ADAE algorithm

ADAE had an AUC of 0.857, which was higher than the AUC of the physician pre-ADAE exposure estimated probability of melanoma (0.780; *p* = 0.007), the lesion maximum diameter (0.758; *p* < 0.001) and patient age (0.649; *p* < 0.001), and similar to the AUC from the histopathologically confirmed 2020 SIIM-ISIC Challenge test set (0.854; *p* = 0.882).

**Fig. 1:**
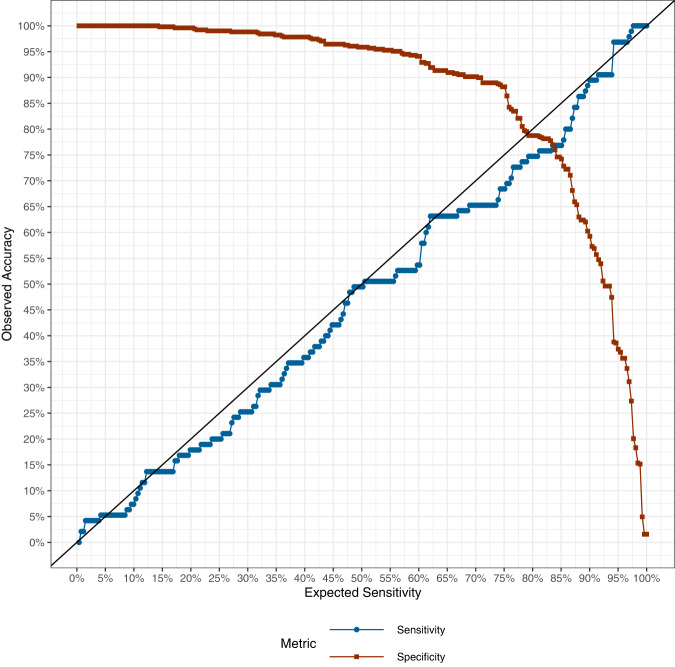
Concordance of expected sensitivity with observed sensitivity. Observed (vertical axis) sensitivity (blue) and specificity (red) at various expected sensitivity thresholds (horizontal axis). A perfect concordance between observed and expected sensitivity is represented by the solid black line. Deviations of the blue line below the black line (observed sensitivity less than expected sensitivity) and above black line (observed sensitivity greater than expected sensitivity) suggest loss of concordance. At the predetermined 95% sensitivity threshold (the study’s prespecified primary endpoint), ADAE had a sensitivity of 96.8% (95% CI: 91.1–98.9%) and specificity of 37.4% (95% CI: 33.3–41.7%).

The concordance between the expected and observed sensitivity across thresholds 0% to 100% was excellent (Fig. 1 and Supplementary Table 3). At the predetermined 95% sensitivity threshold (the study’s prespecified primary endpoint), ADAE had a sensitivity of 96.8% (95% CI: 91.1–98.9%) and specificity of 37.4% (95% CI: 33.3–41.7%). The difference in ADAE sensitivity for invasive vs. in situ melanoma at this threshold was not significant (95.7% vs. 98.0%, *p* = 0.609). The sensitivity for unequivocal melanoma (excluding borderline cases) was 96.3%. Subgroup analyses did not identify patient or lesional factors associated with lower sensitivity, though these comparisons were underpowered. (Table 2).

**Table 2:** Performance of ADAE algorithm at prespecified 95% sensitivity threshold, stratified by various demographic and clinical factors.

Characteristic	Total lesions (n)	Total melanomas (n)	ADAE Sensitivity	ADAE Specificity	ADAE AUC
Overall	603	95	97%	37%	0.858
Age					
<65	349	36	97%	46%	0.867
65+	254	59	97%	24%	0.829
Sex					
Female	324	32	94%	41%	0.806
Male	279	63	98%	32%	0.877
Study dermatologist					
Derm1	389	23	100%	45%	0.822
Derm2	91	26	89%	23%	0.783
Derm3	56	29	100%	0%	0.756
Derm4	28	0	NA	21%	NA
Derm5	22	11	100%	9%	0.926
Derm6–Derm11	17	6	100%	27%	0.924
Lesion location					
Head/neck	66	20	95%	17%	0.863
Torso	288	35	97%	45%	0.858
Upper extremity	117	22	100%	30%	0.913
Lower extremity	127	18	94%	34%	0.746
Palms or soles	5	0	NA	80%	NA
Maximum lesion diameter					
<3.0 mm	49	1	100%	54%	0.625
3.0–6.0 mm	295	22	91%	49%	0.821
>6.0 mm	258	72	99%	17%	0.820
Fitzpatrick skin phototype					
I	46	16	88%	20%	0.746
II	33	57	98%	32%	0.872
III	202	19	100%	48%	0.844
IV	22	3	100%	47%	0.965
Participant nevus phenotype					
Florid	61	16	100%	29%	0.825
Moderate	149	19	100%	41%	0.868
Mild	254	57	100%	40%	0.891
No/few	139	3	91%	30%	0.804
Biopsy reason					
Atypical dermoscopy	459	82	96%	34%	0.858
Change per dermatologist	337	44	93%	42%	0.825
Atypical clinical	328	60	100%	33%	0.906
Ugly duckling/Outlier	124	45	96%	19%	0.821
Change per patient	57	10	100%	34%	0.857
Patient concern/symptoms	37	4	100%	49%	1.000
Atypical RCM	25	8	100%	6%	0.586
Atypical PLA	1	0	NA	100%	NA

Participant nevus phenotype was the dermatologist’s subjective assessment of the number of moles and presence of atypical moles. Dermatologists could select multiple biopsy reasons (non-mutually exclusive). *AUC* area under the receiver operating characteristic curve, *RCM* reflectance confocal microscopy, *PLA* pigmented lesion assay test results.

In multivariate analysis, specificity of the algorithm was significantly associated with patient age, anatomic site, and maximum diameter, being lower in patients 65+ years of age and in lesions on the head/neck and with diameter >6 mm (Table 3). The specificity was also higher in patients with skin-type III than with skin-type I (*p* = 0.002).

**Table 3:** Multivariate analysis of factors associated with ADAE algorithm specificity at prespecified 95% sensitivity threshold.

Characteristic	Total non-melanomas (*N* = 508)	Unadjusted (observed) specificity	Adjusted specificity	95% Confidence Interval	*P*-value
Age					
<65	312	46%	46%	(36–71%)	referent
65+	196	24%	24%	(14–34%)	0.004
Sex					
Female	292	41%	41%	(30–51%)	referent
Male	216	32%	33%	(13–52%)	0.26
Lesion location					
Head/neck	46	17%	18%	(4–32%)	referent
Torso	253	45%	44%	(26–62%)	0.001
Upper extremity	95	30%	30%	(19–40%)	<0.001
Lower extremity	109	34%	34%	(28–41%)	<0.001
Palms or soles	5	80%	80%	(48–112%)	0.05
Maximum lesion diameter					
<=6.0 mm	321	50%	50%	(36–64%)	referent
>6.0 mm	186	17%	18%	(8–28%)	<0.001
Unknown	1				
Fitzpatrick skin phototype					
I	30	20%	22%	(10–35%)	referent
II	276	32%	32%	(21–42%)	0.27
III	183	48%	47%	(39–56%)	0.002
IV	19	47%	47%	(2–92%)	0.28
Participant nevus phenotype					
Florid	45	29%	11%	(9–13%)	referent
Moderate	133	41%	40%	(30–50%)	0.1
Mild	225	40%	40%	(31–50%)	0.15
No/few	105	30%	29%	(15–43%)	0.7
Study dermatologist initial confidence					
1–2	91	25%	25%	(2–49%)	referent
3	80	33%	32%	(12–52%)	0.19
4 (high)	337	42%	42%	(38–46%)	0.07

All models include binary explanatory terms for age and sex.

### ADAE by physician and lesion characteristics

**Fig. 2:**
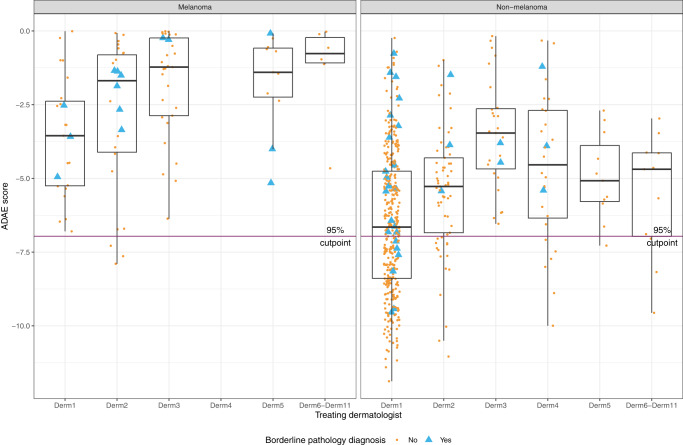
ADAE score distribution by treating dermatologist and pathologic diagnosis. ADAE scores (y-axis) for individual study lesions (yellow dots and blue triangles) are shown for melanomas (left panel) and non-melanomas (right panel), stratified by study dermatologist (x-axis). Higher ADAE scores (more likely melanoma) are closer to 0 and lower ADAE scores (less likely melanoma) are increasingly negative. The horizontal line is the prespecified 95% sensitivity threshold. Yellows dots represent lesions with an unequivocal benign or malignant diagnosis. Blue triangles represent lesions with a borderline pathology that was adjudicated to be melanoma or non-melanoma. The center line within each boxplot represents the median. The lower and upper hinges of the box represent the first and third quartiles (Q1 and Q3). The upper end of each whisker represents the more extreme value between the largest observed value and Q3 + 1.5 * IQR and the lower end of each whisker represents the more extreme value between the smallest observed value and Q1−1.5 * IQR, where IQR is the interquartile range.

The ADAE score distribution on biopsied lesions varied significantly by dermatologist (*p* < 0.001) (Fig. 2). For the 5 dominant dermatologists, the sensitivity of ADAE at the predetermined threshold varied from 89% to 100%. Specificity ranged from 0% to 45%. Eleven (1.8%) lesions were purely amelanotic (1 melanoma, 1 atypical melanocytic proliferation, 1 SCC in situ, and 8 benign lesions). All cases had ADAE scores above the predetermined threshold. Most lesions (*n* = 518, 86%) had surrounding skin with evidence of photodamage. Lesions with photodamage were more likely to be melanoma (16.9% vs. 8.2%; *p* = 0.038 by Fisher’s Exact test). Lesions with vs. without photodamage showed differences in specificity (33.3% vs. 60.3%; *p* < 0.001 by Fisher’s Exact test). The non-melanoma diagnostic classes with the highest proportion of ADAE scores above the predetermined threshold were basal and squamous cell carcinoma (100% each), followed by non-melanocytic collision tumors (94%), actinic keratosis (92%), atypical melanocytic proliferation (89%), lentigo (87%), seborrheic keratosis (74%), and nevus (45%) (Supplementary Table 4).

### Potential effect of recruitment bias

In a separate retrospective study, any skin lesion that underwent biopsy during the study period with a rule out clinical diagnosis including a melanocytic diagnostic term on the pathology requisition order that was not enrolled in the prospective study was identified (*n* = 408). Twenty-five lesions were melanoma and 383 were non-melanoma (Supplementary Table 5). ADAE had an AUC of 0.862 on this dataset (vs. 0.857 in the prospective study, *p* = 0.913). At the predetermined 95% sensitivity threshold, ADAE had a sensitivity of 100% (vs. 96.8%, *p* = 1.00) and specificity of 34.7% (vs. 37.4%, *p* = 0.439). (Supplementary Figs. 3 and 4).

### Potential effect of ADAE exposure on dermatologist decision making

The dermatologists’ pre-ADAE mean confidence was 3.4 (IQR 3–4) and the post-ADAE mean confidence was 3.3 (IQR: 3–4). After considering the ADAE output, confidence increased in 16.4% of cases and decreased in 18.7% of cases. Analysis of the individual dermatologists’ confidence showed that one dermatologist had lower confidence after exposure (OR 0.43, 95% CI: 0.30–0.60, *p* < 0.001), one dermatologist had higher confidence (OR 2.16, 95% CI: 1.3–3.7, *p* = 0.005), and the changes were not significant among the remaining dermatologists (Supplementary Table 6).

The dermatologists’ AUC calculated using their predicted melanoma probabilities improved from 0.7798 to 0.8161 after exposure to ADAE (*p* = 0.042). This improvement persisted after excluding the dermatologist who contributed the most lesions (AUC 0.7663 to 0.8081, *p* = 0.011, calculated using 214 study lesions). The four dominant enrolling dermatologists who biopsied ≥1 melanoma all had an individual improvement in their AUC (range of improvement: +0.0394 to +0.1157) (Supplementary Table 7, Supplementary Fig. 5).

Calibration assesses the agreement between predicted risks and observed outcomes^12^. The mean dermatologist predicted melanoma probability changed from 20% (IQR: 5–20%) to 24% (IQR: 2–40%), indicating worse mean calibration after exposure to ADAE as the overall melanoma prevalence was 16% (*p* < 0.001). The effect varied among the 5 dominant-enrolling dermatologists, however, with 3 improving and 2 worsening their mean calibration (Supplementary Table 7).

After ADAE exposure, dermatologists theoretically decided to avoid skin biopsy in 29% of lesions (*n* = 175), but this individually ranged from 0% to 39%. In 116 cases non-invasive testing (that is, STM, RCM, adhesive patch application) was chosen and in 59 cases no testing (that is, routine follow-up) was chosen (Table 4). The sensitivity and specificity of the biopsy vs. no biopsy decision threshold was 96% and 34% (Table 5); this means that 4 out of 95 melanomas would not have undergone biopsy but that 171 out of 508 benign lesions would have been spared an unnecessary biopsy. Dermatologists chose to biopsy 94% (386 out of 410) of the lesions that scored above the ADAE 95% sensitivity threshold and chose not to biopsy 78% (151 out of 193) of the lesions that scored below the threshold. In addition, of the three melanomas that scored below the 95% sensitivity threshold, dermatologists chose to continue to biopsy two of the melanomas. The sensitivity and specificity of the test vs. no test decision threshold was 98% and 11%.

**Table 4:** Dermatologist clinical management decision after exposure to AI algorithm, stratified by histopathological diagnosis.

Pathologic diagnosis	Total	Biopsy		Short-term monitoring		Reflectance confocal microscopy		Adhesive skin patch testing		Routine follow-up	
Overall	603	428	71%	104	17%	3	0%	9	1%	59	10%
Invasive melanoma	46	42	91%	1	2%	0	0%	1	2%	2	4%
Melanoma in situ	49	49	100%	0	0%	0	0%	0	0%	0	0%
Keratinocyte carcinoma	22	22	100%	0	0%	0	0%	0	0%	0	0%
Atypical melanocytic proliferation	28	23	82%	2	7%	1	4%	1	4%	1	4%
Nevus	312	161	52%	92	29%	2	1%	7	2%	50	16%
Other	146	131	90%	9	6%	0	0%	0	0%	6	4%

**Table 5:** Post-AI exposure biopsy decisions, stratified by dermatologist.

Study dermatologist	Post-AI management sensitivity	Post-AI management specificity
Derm1	100% (23/23)	38.5% (141/366)
Derm2	84.6% (22/26)	30.1% (20/65)
Derm3	100% (29/29)	0% (0/27)
Derm4	N/A (0/0)	28.6% (8/28)
Derm5	100% (11/11)	0% (0/11)
Derm6-11	100% (6/6)	18.2% (2/11)
Overall	95.8% (91/95)	33.7% (171/508)

### Clinical utility

**Fig. 3:**
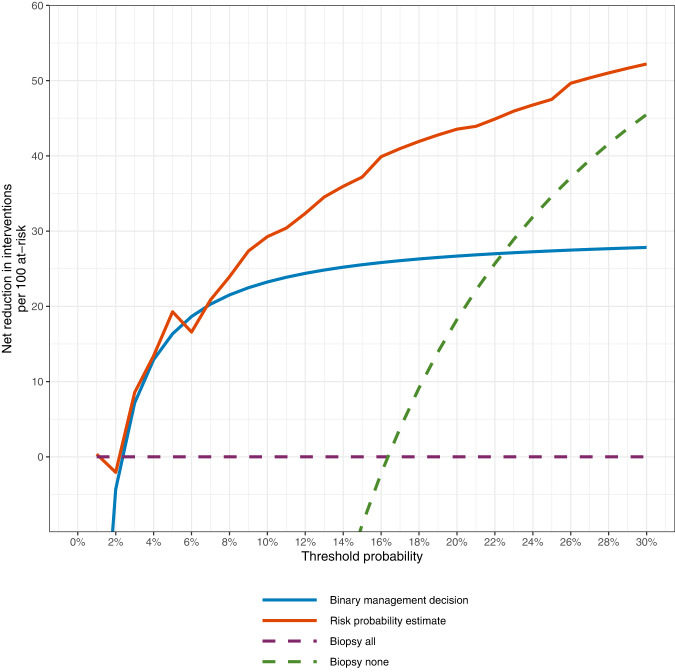
Decision curve plotting decrease in avoidable interventions against threshold probability. The binary management decision is the dermatologist biopsy decision (yes/no) after exposure to AI results. The risk probability estimate is the dermatologist’s predicted melanoma probability (0–100%) after exposure to AI results. The default strategy (biopsy all lesions) is represented by the x-axis (net avoidable interventions of 0). For example, at a threshold probability of 5% (meaning that harm of missing 1 melanoma is equivalent to the harm of 19 unnecessary benign skin biopsies), exposing dermatologists to ADAE results would theoretically be the equivalent of a strategy that reduced the number of unnecessary biopsies by about 15–20 per 100 without missing biopsy for any patients with melanoma. All 22 histopathologic keratinocyte carcinomas were excluded from decision curve analyses because they are not viewed as equivalent to benign skin lesions.

At relevant threshold probabilities for a skin biopsy to rule out melanoma (defined broadly as 2–20%), post-ADAE exposure dermatologist decisions (biopsy decision and post-ADAE predicted melanoma probability) had higher or equivalent net benefit compared to the baseline strategy of biopsying all lesions. In clinical terms, this improvement can also be conceptualized as a net positive reduction in avoidable interventions (biopsies without a melanoma diagnosis, after accounting for the harms of missed melanomas) (Fig. 3).

## Discussion

We prospectively assessed the diagnostic accuracy and potential utility of a melanoma AI algorithm in a specific intended use clinical setting: lesions selected by a dermatologist for biopsy to rule out melanoma. Furthermore, factors associated with diagnostic performance were characterized. The sensitivity of ADAE was found to be consistent after considering patient- and lesion-related factors. However, ADAE specificity was lower in patients 65+ years of age and those with skin-type I. Specificity was also lower in lesions on the head/neck, with a diameter >6 mm, and with surrounding perilesional photodamage, and in actinic keratoses, solar lentigines, and non-melanocytic collision tumors. These findings may help identify clinical scenarios in which ADAE has the greatest potential utility or requires further training. Based on these results, we hypothesize that ADAE may be most helpful when challenged with a suspicious lesion on the trunk or extremities in a younger individual with a clinical differential diagnosis of nevus. It may be comparatively less helpful when assessing older individuals with photo-damage with a clinical differential diagnosis of solar lentigo. Randomized clinical trials are required to formally assess the potential benefits and harms of providing dermatologists with ADAE results after identifying a skin lesion suspicious for melanoma.

Compared to retrospective studies, use of a prospective design may have reduced the effect of certain biases on diagnostic accuracy estimates, including biases affecting patient selection and disease spectrum, diagnostic review (reference and index test results are not independently interpreted), differential verification (using two different reference standards), and inconclusive results (how borderline reference or index test results are handled)^13^. The prospective design may have mitigated the effect of other factors noted to limit retrospective studies of skin cancer AI, including the Clever Hans bias^14^ and the lack of out-of-distribution cases^15^. Retrospective studies may also preferentially select cases with images of sufficient or optimized quality, or with the most diagnostic features.

A unique aspect of this study was the comparison of the diagnostic accuracy of ADAE to expert dermatologists practicing in a real-world clinical setting. Prior studies have been limited by using reader-based methodology in experimental settings with incomplete clinical information, by focusing on the performance of residents/trainees or teledermatologists, or by incompletely assessing the performance of dermatologists^4^. We showed that the discrimination of ADAE was significantly higher than that of dermatologists whose clinical expertise is skin cancer detection. ADAE performance was also superior to readily available and objective clinical predictors of melanoma diagnosis, like lesion size and patient age. Furthermore, exposure to ADAE results consistently improved all dermatologists’ ability to distinguish melanoma from benign mimickers as measured by AUC. The effect of ADAE on dermatologists’ calibration, confidence, and theoretical management decisions was inconsistent. This may be partially due to dermatologists’ unfamiliarity with estimation of melanoma probabilities and variable trust in AI performance. Unlike prior studies, we provided a more granular and transparent display of AI results, including spatial support for diagnostic class prediction.

Exposure to ADAE theoretically changed the dermatologists’ management decision in nearly one-third of cases, although this varied by individual dermatologist. The potential clinical impact was assessed using decision curve analysis, which considers the trade-off of avoiding unnecessary biopsies at the expense of omitting a skin biopsy of a melanoma^16–18^. At risk thresholds of 2% and above, meaning a dermatologist is not willing to perform more than 49 unnecessary skin biopsies to identify 1 melanoma, the dermatologists’ post-ADAE exposure management choices had equivalent or higher net benefit than the default strategy of biopsy all lesions. Only if a dermatologist is willing to perform 50 or more unnecessary skin biopsies to identify 1 melanoma would biopsying all lesions have higher net benefit.

Study limitations include its single-center design, inclusion of few dermatologists, and relatively small sample size of lesions, particularly rare melanoma subtypes. These are characteristics of smaller pilot studies, such as the one we performed, which are necessary prerequisites to larger multicenter studies. These limitations affect both the validity and generalizability of our data. We also did not biopsy any lesions in patients with Fitzpatrick Skin Type V or VI during the study period, and the accuracy of ADAE in this population requires further study. A multicenter study that draws large numbers of patients from diverse populations would build upon the results of this study and overcome its limitations. Due to time, funding, patient care, and staff constraints, not all eligible participants were approached for study inclusion, which may have led to recruitment bias. To specifically study the effect of recruitment bias, we retrospectively identified non-enrolled lesions and did not identify a significant difference in diagnostic accuracy estimates. Furthermore, we did not study non-biopsied skin lesions nor biopsied melanomas not considered to be potential melanomas, as these lesions are currently outside of the intended use of this algorithm. A unique strength of our study is that we assessed an open-source, non-commercial algorithm and that all our study images have been made freely available through the International Skin Imaging Collaboration’s online image archive. The public availability of our data set will permit external analyses and benchmarking against other AI algorithms. Additional prospective studies at other medical centers are ultimately needed to further validate the transportability of ADAE. Randomized trials are needed to evaluate the effects of adopting this AI algorithm into clinical workflows.

## Methods

All research was approved by the Institutional Review Board at Memorial Sloan Kettering Cancer Center (MSKCC) under IRB 21-019 and adhered to the Helsinki Declaration. Here, we provide more details on each individual study (study 1—prospective validation of ADAE diagnostic accuracy; study 2—potential effect of ADAE on dermatologist decision making).

### Algorithm description and rationale

The Society for Imaging Informatics in Medicine—International Skin Imaging Collaboration (SIIM-ISIC) 2020 Melanoma Classification challenge was hosted by Kaggle and used a convenience test set of 10,982 public dermoscopy images from 6 dermatology centers (Barcelona, Spain; New York, United States; Vienna, Austria; Sydney, Australia; Brisbane, Australia; Athens, Greece)^19^. ADAE was the top-ranked algorithm of 3,308 competing teams, with an overall AUC of 0.9490, and was chosen for this study^20^. ADAE uses a set of 18 prediction models (each trained on 5 different folds for validation, for a total set of 90 model weight files). Sixteen models are based on EfficientNet architecture and two use ResNet^21^ architecture^22–24^. Four models use clinical metadata (age, sex, anatomic site, and image size). The models were combined as in the original submission to the 2020 Melanoma Classification challenge except the 90 individual fold scores were log-transformed prior to averaging.

### Combining and cross-model ensembling of scores

The post-softmax values for the 4 (rotation) by 2 (flipping state) images passed through a given model are first averaged. This score is computed for batches of images, until all scores for the full set of images have been computed. We updated the Kaggle submission winner by implementing a log-transform for the 90 individual fold scores prior to averaging. Over the 2020 challenge data, the raw outputs aggregation and log-transformed outputs aggregation attained AUCs of 0.9492 and 0.9502, although these were not significantly distinguishable (*p* = 0.4177; DeLong’s test). ADAE usage notes are provided in the suppementary file.

To further support the rationale to study ADAE in a patient setting prior to initiation of the current study, we validated its performance on prior ISIC Melanoma Classification challenge test datasets. ADAE exceeded the performance of the top-ranked algorithms from the 2019 (AUC: 0.970 vs. 0.949) and 2018 (AUC: 0.932 vs. 0.928) challenges. We also retrospectively retrieved images from 147 consecutive biopsies (46 melanomas and 101 non-melanomas) to rule out melanoma performed in 2020 from a high-risk screening clinic at Memorial Sloan Kettering Cancer Center. Using the predetermined algorithm cutoff associated with 95% sensitivity in the SIIM-ISIC 2020 challenge test set, ADAE had a sensitivity of 93% (95% CI: 85–98%) and specificity of 25% (95% CI: 17–34%) suggesting temporal validity of the prediction model.

### Study 1. Accuracy of the ADAE algorithm design and participant recruitment

We assessed the accuracy of ADAE in two ways. First, we conducted a prospective, single-arm, observational clinical validation study to assess the diagnostic accuracy of ADAE in predicting melanoma from dermoscopy skin lesion images. The hypothesis was that ADAE would correctly identify ≥90% of biopsied skin melanomas using a predefined threshold corresponding to 95% sensitivity on the multi-institutional test set curated for the 2020 SIIM-ISIC Melanoma Classification challenge. Patients who had consented for a skin biopsy of a lesion to exclude cutaneous melanoma (as determined by their dermatologist) were eligible. Participants ≥18 years of age were enrolled from dermatology clinics in New York and New Jersey from September 30, 2021, to June 24, 2022. Previously biopsied, recurrent, and mucosal lesions, as well as lesions removed for cosmetic purposes, were not eligible. Reporting of data followed the STARD checklist.

Second, to investigate potential recruitment bias on estimates of ADAE’s accuracy in the prospective study (that is, dermatologists not enrolling melanomas judged to be at low risk of being melanoma), we retrospectively identified any skin lesion that underwent biopsy during the study period with a rule out clinical diagnosis including a melanocytic diagnostic term on the pathology requisition order that were not enrolled in the prospective study. This analysis was limited to dermatologists consenting ≥20 participants to the prospective study.

### Study 1 Data collection

Per institutional standard of care, all skin lesions subjected to biopsy undergo clinical and dermoscopic (polarized and nonpolarized) imaging. For the prospective diagnostic accuracy study, a study member uploaded in real-time (either prior to the biopsy or immediately thereafter) the lesional dermoscopy image assessed by the treating dermatologist to be most clinically representative along with clinical metadata (participant age, participant self-reported sex, and lesion anatomic site) to a dedicated study web-app. After upload, the lesion’s ADAE score was computed and automatically stored in a secured study database. No reference standard results were available when the index test (ADAE) was calculated. Skin biopsy specimens were submitted for pathology interpretation per standard of care. Dermatopathologists (reference standard assessors) were blinded to ADAE results but exposed to the standard clinical information provided on pathology requisitions. Pathology reports were later reviewed by a dedicated and trained study member and categorized by diagnosis. Lesions with an equivocal diagnosis (for example, atypical melanocytic proliferation) were separately categorized as borderline lesions. Pathology reports of borderline lesions were independently reviewed by two dermatologists blinded to clinical metadata and index test results and categorized as either “melanoma” or “not melanoma”. Discrepancies were adjudicated by a third dermatologist. Dermoscopy images were independently reviewed by two trained study members for the presence or absence of perilesional photodamage (telangiectasias and/or lentigines) and lesional melanotic status (purely amelanotic or not). Discrepancies were adjudicated by a study dermatologist.

To identify recruitment bias, eligible pathology reports of non-enrolled biopsies were reviewed in the identical manner as the prospective study and categorized by diagnosis. For each lesion, the contact polarized dermoscopy image was obtained from the medical chart. ADAE scores were computed in the identical manner as the prospective study (including patient age, sex, and anatomic site). Reference standard results were available when ADAE was calculated.

### Study 1 Sample size calculation

The primary aim was to assess the reliability of ADAE’s sensitivity for melanoma classification on prospectively acquired cases at a predefined threshold corresponding to 95% sensitivity on a multi-institutional dataset curated for the 2020 SIIM-ISIC Melanoma Classification challenge. We defined a non-inferiority test with an acceptable margin no greater than 5%. Through a Monte Carlo random sampling approach, it was determined that 86 melanoma cases would power the study at 80% to demonstrate a true-positive fraction greater than 90% if the parametric sensitivity is indeed 95%. Eighty-six melanomas were estimated to be a reasonable target across 9-months of accrual based on the frequency of melanoma diagnosed at the institution in recent years. Outcome definitions are provided in Supplementary Table 1.

### Study 1 Statistical analysis

ADAE score was analyzed both as a continuous variable and a binary classification using a predetermined threshold as outlined above. Uncertainty in binary classification accuracy was estimated using Wilson score intervals. Fisher’s exact test was used to determine if associations between categorical factors and binary outcomes were statistically significant. ADAE specificity was estimated with binomial generalized linear models (GLM) with a log link and cluster-robust standard errors to account for clustering between cases enrolled by the same dermatologist. GLMs contained fixed effects to account for patient age and sex.

ADAE discrimination was visualized with ROC curves and summarized with AUC. DeLong’s test for uncorrelated ROC curves was used for subgroup analyses. DeLong’s test for correlated ROC curves was used to compare ADAE discrimination to age and lesion size, and when comparing performance on dermoscopy image types. ADAE score distributions between subgroups were compared with Kruskal-Wallis tests. The chosen level of significance was 0.05, and analyses were performed in R (R Core Team (2022). R: A language and environment for statistical computing. R Foundation for Statistical Computing, Vienna, Austria. URL https://www.R-project.org/.) and STATA (v16.1)

### Study 2. Potential utility of ADAE algorithm in dermatologist decision making

Prior decision-impact studies have been limited by being conducted outside of a clinical setting. In these experimental reader studies, dermatologists have assessed images of lesions from patients, generally with limited or no knowledge of clinical metadata. To better understand the real-time potential impact of exposure to AI on dermatologists, we conducted a theoretical decision-impact study in parallel to the prospective validation study previously described. The study hypothesis was that exposure to ADAE algorithm scores will improve dermatologists’ theoretical diagnostic predictions and management choices of their own patients. All patients who consented to the prospective accuracy study were included. Dermatologists provided written informed consent prior to participation.

### Study 2 Data collection

**Fig. 4:**
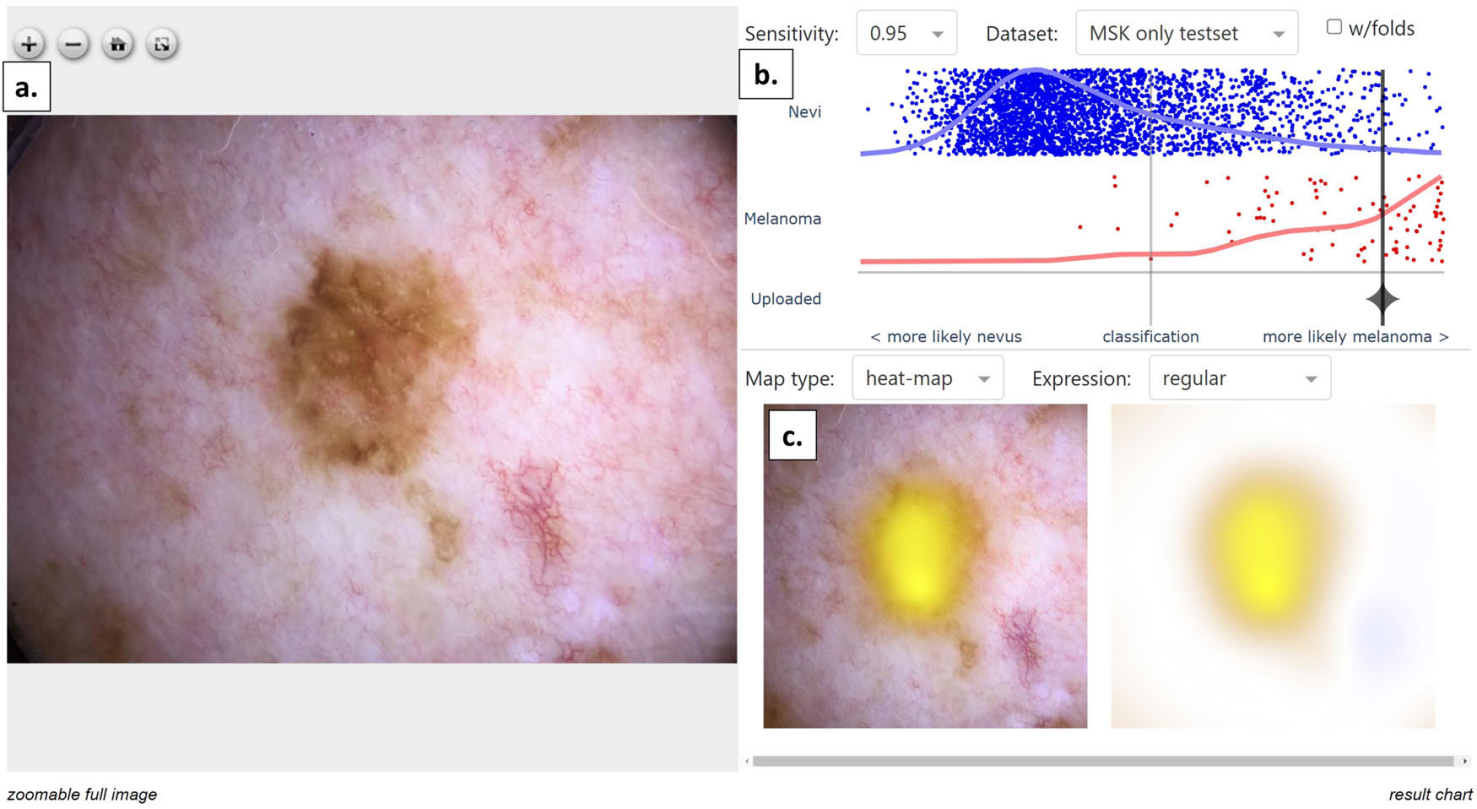
ADAE web-app interface. Example of image upload results. **a** Dermoscopy image chosen by the dermatologist. **b** The ADAE score is shown as the vertical black line, calculated as the log-average of the 18 models (90 folds). Red (melanoma) and blue (benign lesions) dots were from the 2020 SIIM-ISIC Melanoma Challenge, with scores calculated as the average of the 18 models (90 folds). Higher scores (more likely melanoma) were to the right and lower scores (more likely benign) were to the left. The horizontal red (melanoma) and blue (benign lesions) lines show the distributions of ADAE scores for these diagnostic classes. Scores were spline transformed so the 95% sensitivity of the raw average of the 18 models was displayed in the center (gray vertical line). Users could adjust the interface to visualize different sensitivity thresholds (95%, 90%, 85%, and 80%) and different data test sets (red and blue dots) from the SIIM-ISIC Melanoma Challenge (MSK only vs. all 6 sites). **c** Saliency map showing the spatial support for melanoma prediction with yellow color indicating more likely melanoma and blue color indicating more likely not melanoma.

After consenting a study participant, the dermatologist completed a pre-AI exposure survey. The survey included participant skin type, nevus phenotype, personal history of melanoma, family history of melanoma, reason for biopsy, and differential diagnosis. In addition, the dermatologist specified their pre-AI estimated probability of melanoma (0–100%) and their confidence in their estimated melanoma probability [1(low)-4(high)]. Subsequently, a study member uploaded in real-time (either prior to biopsy or immediately thereafter) the lesional dermoscopy image assessed by the treating dermatologist to be most clinically representative. Dermoscopy images were captured by a trained technologist across all practice sites. The ADAE score was generated within 1-minute and visually displayed on the web-app to the dermatologist (Fig. 4). After viewing the ADAE score, the dermatologist recorded a post-AI estimated probability of melanoma (0–100%), confidence in their estimated melanoma probability [1(low)-4(high)], and new theoretical management choice [Q:If you could change your management, what would you do now? (select one); choices: I would still biopsy, STM, RCM, PLA, routine follow-up]. Patients were not exposed to the AI scores of their lesions and all lesions underwent biopsy per standard of care.

### Study 2 Statistical analysis

The effect of ADAE on confidence was analyzed using dermatologist-specific ordinal logistic regression models, with response being their indicated confidence and with predictor being whether the response came before or after viewing ADAE. DeLong’s correlated test of ROC curves was used to compare discrimination [AUC, 0.5 (random) to 1 (perfect)] before and after ADAE exposure. The distribution of melanoma-probability estimation was compared before and after ADAE exposure using Kolmogorov–Smirnov test. Clinical utility was explored using decision curve analysis^16^; keratinocyte carcinoma was excluded from this analysis as the benefits and harms of biopsying keratinocyte carcinoma are not equivalent to melanoma or benign skin lesions.

### Reporting summary

Further information on research design is available in the Nature Research Reporting Summary linked to this article.

## Supplementary information

Supplementary Materials

Reporting Summary

## Data Availability

All dermoscopy images analyzed for the patients who consented for the prospective study are freely available through the International Skin Imaging Collaboration’s online image archive at 10.34970/576276.

## Code availability

The code to run inference on the pretrained ADAE algorithm is available on github https://github.com/ISIC-Research/ADAE.

## References

[1] Nelson KC, Swetter SM, Saboda K, Chen SC, Curiel-Lewandrowski C (2019). Evaluation of the number-needed-to-biopsy metric for the diagnosis of cutaneous melanoma: a systematic review and meta-analysis. JAMA Dermatol..

[2] Fried L (2020). Technological advances for the detection of melanoma: Advances in diagnostic techniques. J. Am. Acad. Dermatol..

[3] Fried L (2020). Technological advances for the detection of melanoma: Advances in molecular techniques. J. Am. Acad. Dermatol..

[4] Haggenmüller S (2021). Skin cancer classification via convolutional neural networks: systematic review of studies involving human experts. Eur. J. Cancer.

[5] Daneshjou R (2022). Checklist for Evaluation of Image-Based Artificial Intelligence Reports in Dermatology: CLEAR Derm Consensus Guidelines From the International Skin Imaging Collaboration Artificial Intelligence Working Group. JAMA Dermatol..

[6] Daneshjou R, Smith MP, Sun MD, Rotemberg V, Zou J (2021). Lack of Transparency and Potential Bias in Artificial Intelligence Data Sets and Algorithms: A Scoping Review. JAMA Dermatol..

[7] Combalia M (2022). Validation of artificial intelligence prediction models for skin cancer diagnosis using dermoscopy images: the 2019 International Skin Imaging Collaboration Grand Challenge. Lancet Digit Health.

[8] Marchetti MA (2018). Results of the 2016 International Skin Imaging Collaboration International Symposium on Biomedical Imaging challenge: Comparison of the accuracy of computer algorithms to dermatologists for the diagnosis of melanoma from dermoscopic images. J. Am. Acad. Dermatol..

[9] Marchetti MA (2020). Computer algorithms show potential for improving dermatologists' accuracy to diagnose cutaneous melanoma: Results of the International Skin Imaging Collaboration 2017. J. Am. Acad. Dermatol..

[10] Rotemberg V (2021). A patient-centric dataset of images and metadata for identifying melanomas using clinical context. Sci. Data.

[11] Tschandl P (2019). Comparison of the accuracy of human readers versus machine-learning algorithms for pigmented skin lesion classification: an open, web-based, international, diagnostic study. Lancet Oncol..

[12] Van Calster B, McLernon DJ, van Smeden M, Wynants L, Steyerberg EW (2019). Calibration: the Achilles heel of predictive analytics. BMC Med..

[13] Schmidt RL, Factor RE (2013). Understanding sources of bias in diagnostic accuracy studies. Arch. Pathol. Lab Med.

[14] Lapuschkin S (2019). Unmasking Clever Hans predictors and assessing what machines really learn. Nat. Commun..

[15] Han SS (2022). Evaluation of Artificial Intelligence-Assisted Diagnosis of Skin Neoplasms: A Single-Center, Paralleled, Unmasked, Randomized Controlled Trial. J. investigative Dermatol..

[16] Vickers AJ, Van Calster B, Steyerberg EW (2016). Net benefit approaches to the evaluation of prediction models, molecular markers, and diagnostic tests. BMJ (Clin. Res. ed.).

[17] Kerr KF, Brown MD, Zhu K, Janes H (2016). Assessing the Clinical Impact of Risk Prediction Models With Decision Curves: Guidance for Correct Interpretation and Appropriate Use. J. Clin. Oncol. : Off. J. Am. Soc. Clin. Oncol..

[18] Fitzgerald M, Saville BR, Lewis RJ (2015). Decision curve analysis. Jama.

[19] SIIM-ISIC Melanoma Classification. Accessed November 9, 2022. https://www.kaggle.com/c/siim-isic-melanoma-classification.

[20] Grandmaster Series – How to Build a World-Class ML Model for Melanoma Detection. Accessed November 9, 2022. https://developer.nvidia.com/blog/grandmaster-series-how-to-build-a-world-class-ml-model-for-melanoma-detection/.

[21] Szegedy C., Ioffe S., Vanhoucke V., Alemi A. Inception-v4, Inception-ResNet and the Impact of Residual Connections on Learning. *Proceedings of the AAAI Conference on Artificial Intelligence*. 31 10.1609/aaai.v31i1.11231 (2017)

[22] Gessert N, Nielsen M, Shaikh M, Werner R, Schlaefer A (2020). Skin lesion classification using ensembles of multi-resolution EfficientNets with meta data. MethodsX.

[23] Identifying Melanoma Images using EfficientNet Ensemble: Winning Solution to the SIIM-ISIC Melanoma Classification Challenge. Accessed November 9, 2022. 10.48550/arXiv.2010.05351.

[24] Tan, M. & Le, Q. V. EfficientNet: Rethinking Model Scaling for Convolutional Neural Networks. *ArXiv*. 2019;abs/1905.11946.

