# Supplementary material for: Prospective validation of dermoscopy-based open-source artificial intelligence for melanoma diagnosis (PROVE-AI study)
Source: NPJ Digit Med. 2023 Jul 12;6:127. doi: 10.1038/s41746-023-00872-1 (PMC10338483; doi:10.1038/s41746-023-00872-1)

Supplementary Materials

Methods:

ADAE algorithm usage notes

Code and models: The "ADAE model" was implemented using python and pytorch, based on generic EfficientNet (geffnet) and ResNeSt-101 (https://arxiv.org/abs/2004.08955) architectures.[15-17] The ensemble of models consists of a total of 18 separate models (16 EfficientNet, 2 ResNeSt-101), each having been trained on 5 folds of data (with 20% of the total training set being held out for internal validation), meaning that to generate scores for images, a total of 90 models need to be evaluated. The image size passed through the 18 separate models ranges from 384-by-384 pixels to 896-by-896 pixels. Each of the models produces an internal "feature" representation of the image (average pooling the last convolutional layer across pixels to a single value per feature map). The number of features ranges from 1,536 to 2,560 for the EfficientNet models, and is set to 2,048 for the ResNeSt-101 models. Each of the models then uses a final fully connected layer to predict a diagnosis class label from this high-dimensional feature space.

Data Augmentation: Each image has its central square portion cut out (i.e. the parts outside the central square are discarded) to avoid scaling (shrinking) in only one of two image dimensions. The central square portion is scaled to one of three resolutions (512, 768, or 1024 pixels on both sides), which then allows for more efficient processing through the various models. In addition, each image (square portion) is passed through each of the 90 total folds in 4 rotations x 2 flipped states (unflipped and flipped), to further stabilize scores.

Metadata: Four (4) of the EfficientNet models also expect the following metadata fields: (1) age (which for the training and original test data of the challenge was binned into 5-year brackets to prevent PHI leakage), (2) sex (as 'female', 'male', or 'unknown'), (3) broad anatomic location ('head/neck', 'oral/genital', 'upper extremity', 'palms/soles', 'torso', 'anterior torso', 'posterior torso', 'lateral torso', 'lower extremity', 'unknown'), and (4), derived from patient identifiers, the number of images per patient, as well as (5) extracted image width and (6) image height (of the original image prior to central square extraction and resizing). Sex and anatomic location are dummy-coded into 3 and 10 separate binary variables.

Internal prediction mechanism: The fully connected layer (head) at the end of the model processing chain produces unbound values (between -Infinity and +Infinity). To normalize these scores, the softmax algorithm (see https://en.wikipedia.org/wiki/Softmax_function) is applied across the number of output classes, leading to a set of values between 0 and 1, summing up to 1 for each given image. Seventeen of the 18 models internally predict a class label from among nine (9) target classes (AK = actinic keratosis, BCC = basal cell carcinoma, BKL = benign keratosis, DF = dermatofibroma, MEL = melanoma, NV = nevus, SCC = squamous cell carcinoma, VASC = vascular lesion, UNK = unknown diagnosis), with the remaining model internally predicting a class label from among four (4) target classes (BKL, MEL, NV, and UNK). For the purpose of the challenge, the submitting team chose to simply extract the (post-softmax) value for the MEL (melanoma) output class.

Combining and cross-model ensembling of scores:

Original submission: the post-softmax values for the 4 (rotation) by 2 (flipping state) images passed through a given model are first averaged. This score is computed for batches of images, until all scores for the full set of images have been computed. Next, the 5 folds of a given model are averaged [in the original submission uploaded to Kaggle, due to a coding error, only the 5th/last fold of each model was used for the ensembling; the AUC reported on the Kaggle competition site, despite being the winning algorithm, still improves significantly by using all 90 folds]. For the cross-model ensembling, the model authors chose to rank-transform the 18 lists of scores (for, say, the training set), which yields a list of equi-distant scores between 0 and 1 for all images. The final score of any given image (in a set of images) is then computed by averaging these 18 rank-transformed scores into a value between 0 and 1.

Present study: As the need arose to score individual images, we found that averaging the raw (post-softmax) scores across the 90 folds yielded sub-optimal results. The reason the original submission authors chose to rank-transform the data was to improve (reduce) the noise across the spectrum of values. Given the high imbalance in both the training and test data (approximately 3% of images had a melanoma (positive) class label), and the fact that the challenge was scored on AUC (rather than a 50% a priori threshold and accuracy at that threshold), the models generally produce low absolute values, even for positive cases. For instance, across the entire dataset, the raw-score-average scores yielded a threshold of 0.005356 for a 95% sensitivity, corresponding to an average rank/percentile of 0.658. This leads to an artifact by which even one large value of a single model fold (of 0.5 or greater) would push the average above the 95% sensitivity threshold. To avoid this, the authors of the present study decided to implement a log-transform for the 90 individual fold scores prior to averaging. Over the 2020 challenge data, the

raw outputs aggregation and log-transformed outputs aggregation attained AUCs of 0.9492 and 0.9502, although these were not significantly distinguishable (p=0.4177; DeLong's test).

Results:

Lesions were most frequently on the torso (n=288, 48%), followed by the lower extremity (n=127, 21%), upper extremity (n=117, 19%), head or neck (n=66, 11%), and palms or soles (n=5, 1%). A plurality of lesions (n=285, 47%) were 3-5·9mm in maximum diameter, followed by 6mm or greater (n=271, 45%) and 0·1-2·9mm (n=49, 8%).

The most common reasons for biopsy (non-exclusive) were atypical dermoscopy (n=459, 76%) or clinical features (n=328, 54%), change noted by the dermatologist on total body photography and/or dermoscopy (n=337, 56%) and the ugly duckling sign (n=124, 21%). Other factors contributing to biopsy included patient-reported change (n=57, 9%), concern (n=29, 5%), or symptoms (n=10, 2%), and abnormal diagnostic test results with RCM (n=25, 4%) or adhesive patch application (n=1, 0%).

Most melanomas were greater than 6mm in maximum diameter (n=72, 76%) whereas most non-melanomas were less than or equal to 6mm (n=321, 63%). Specificity at the predetermined threshold was 50% in the 344 lesions less than or equal to 6 mm compared to 17% in the 258 lesions greater than 6 mm (p<0·001). No difference in sensitivity was observed (91% vs. 99%, p=0·144).

We compared the AUC of the full 18 model ADAE with the 14 models that did not incorporate clinical metadata and found no significant difference. The AUC achieved by the 18-model ensemble was 0.857 and the ensemble of 14-models that did not utilize metadata achieved 0.860 (P-value = 0.411; DeLong's test for two correlated ROC curves).

Potential effect of image choice: Most images were captured using the Canfield Scientific (Parsippany, NJ) Veos DS3^{TM} system, but dermatologists used what was the standard practice in their clinic, which could also include the Canfield Veos SLR. In most cases a contact polarized dermoscopy image was chosen as the clinically most representative image (88%, after excluding 79 unknowns); Derm1 = 95%, Derm2 = 61%, Derm3 = 94%, Derm4 = 83%, Derm5 = 20%, Derm6-11 = 91%. No difference was identified in the AUC of ADAE between the image uploaded to the study web-app and a randomly selected dermoscopy image from the remaining unselected images (0·857 v. 0·861, p=0·740). Additionally, no differences in ADAE AUC were found on contact polarized, contact non-polarized, and non-contact polarized images on the subset of lesions (n=526) with all 3 image types available (**Figures S1-2**). We did not find a significant difference in AUC between contact polarized images (0.869 [95%CI (DeLong): 0.823-0.914]) and contact non-polarized images (0.865 [95% CI: 0.815-0.915]) (p=0.715), nor with non-contact polarized images (0.857 [95% CI: 0.817-0.907]) (p=0.343). At the predetermined 95% sensitivity threshold, the sensitivity and specificity of ADADE was 97% and 40% on contact polarized images, 96% and 37% on contact nonpolarized images, and 97% and 34% on non-contact polarized images. However, ADAE score distributions were not equivalent across the 3 image types (p=0.001), suggesting a domain shift.

Effect of ADAE on dermatologist calibration: After exclusion of the dermatologist who contributed the most study lesions, the mean dermatologist predicted probability improved from 29.8% to 32.1% (33.6% mean prevalence). However, this difference was not significantly different (p=0.890).

Supplementary Table 1. Definitions of study outcomes.

Performance metric	Equation
Sensitivity	$\frac{\text{True Positives}}{\text{True Positives } + \text{ False Negatives}}$
Specificity	$\frac{\text{True Negatives}}{\text{True Negatives } + \text{ False Positives}}$
Net benefit	$\frac{\text{True Positives}}{\text{Population}} - \frac{\text{False Positives}}{\text{Population}} * \frac{\text{Risk Threshold}}{1 - \text{Risk Threshold}}$
Net avoidable biopsies	$\frac{\text{True Negatives}}{\text{Population}} - \frac{\left(\frac{\text{False Negatives}}{\text{Population}}\right)}{\left(\frac{\text{Risk Threshold}}{1 - \text{Risk Threshold}}\right)}$
Number needed to biopsy	$\frac{\text{True Positives} + \text{False Positives}}{\text{True Positives}}$

Supplementary Table 2. Histopathological diagnoses of study lesions.

Histopathologic Diagnosis	Count
Melanoma	95
Invasive Melanoma	46
Melanoma in situ	49
Non-melanoma	508
AMP	28
NOS	22
Collision	6
with AK and lentigo	1
with angioma	1
with lentigo	2
with lentigo and SK	1
with SK	1
Keratinocyte carcinoma	22
BCC	9
High-risk	1
Low-risk	8
NOS	7
collision with SK	1
SCC in situ	13
NOS	9
Collision	4
with DN, mod	1
with nevus, congenital	1
with SK and lentigo	2
Nevus	312
with atypia	254
High-grade	69
DN, mod-severe	49
DN, mod-severe and congenital	1
DN, severe	19
Low-grade	185
DN	9
DN and lichenoid keratosis	1
DN, mild	49
DN, mild and congenital	2
DN, mild and verrucous keratosis	1

		DN, mild-mod	35
		DN, mod	76
		DN, mod and congenital	1
		DN, mod and SK	2
		Nevus, atypical	7
		Nevus, atypical and lentigo	1
		Nevus, mild	1
	Nevus other		58
		Blue	1
		Combined	4
		Congenital	3
		Recurrent	2
		Sclerosing	1
		Spitz	1
		Traumatized	1
		Nevus NOS	39
		Collision with lentigo	1
		Collision with lichenoid keratosis	4
		Collision with SK	1
Other			146
	Acantholytic acanthoma		1
	AK		13
	Angiofibroma		1
	Dermatofibroma		9
	Lentigo		45
	Atypical lentigo		2
	Lichenoid keratosis		16
	Lobular capillary hemangioma		1
	Scar		1
	SK		23
	Verrucous keratosis		2
	Collision		32
		with AK and lentigo	4
		with AK and SK	2
		with dermatofibroma and lentigo	1
		with dermatofibroma and SK	1
		with lentigo and dermal hemosiderin	1
		with lentigo and SK and AK	2
		with lichenoid keratosis and lentigo	6
		with lichenoid keratosis and melanocyte hyperplasia	1
		with SK and lentigo	13
		with SK and lentigo and lymphohistiocytic infiltrate with eosinophils	1

Supplementary Table 3. ADAE performance at various expected sensitivity thresholds.

95% CI: 95% Confidence Interval (Wilson method); TP: true positives; FN: false negatives; TN: true negatives; FP: false positives.

	Overall prospectively accrued cases								Sensitivity		Unequivocal cases	
**Expected Sensitivity**	**Sensitivity (95% CI)**		**Specificity (95% CI)**		**TP**	**FN**	**TN**	**FP**	**Invasive mm (N=46)**	**In-situ mm (N=49)**	**Sensitivity (N=81)**	**Specificity (N=476)**
100%	100.0%	(96.1%, 100%)	1.6%	(0.8%, 3.1%)	95	0	8	500	100.0%	100.0%	100.0%	1.7%
95%	***96.8%***	***(91.1%, 98.9%)***	***37.4%***	***(33.3%, 41.7%)***	***92***	***3***	***190***	***318***	***95.7%***	***98.0%***	***96.3%***	***38.7%***
90%	89.5%	(81.7%, 94.2%)	59.3%	(54.9%, 63.4%)	85	10	301	207	87.0%	91.8%	87.7%	60.3%
85%	76.8%	(67.4%, 84.2%)	74.2%	(70.2%, 77.8%)	73	22	377	131	73.9%	79.6%	75.3%	75.0%
80%	74.7%	(65.2%, 82.4%)	78.7%	(75%, 82.1%)	71	24	400	108	69.6%	79.6%	74.1%	79.8%
75%	68.4%	(58.5%, 76.9%)	88.2%	(85.1%, 90.7%)	65	30	448	60	65.2%	71.4%	69.1%	89.1%
70%	65.3%	(55.3%, 74.1%)	90.2%	(87.3%, 92.5%)	62	33	458	50	60.9%	69.4%	65.4%	91.0%
65%	63.2%	(53.1%, 72.2%)	90.9%	(88.1%, 93.1%)	60	35	462	46	56.5%	69.4%	63.0%	91.6%
60%	53.7%	(43.7%, 63.4%)	94.1%	(91.7%, 95.8%)	51	44	478	30	52.2%	55.1%	54.3%	94.7%
55%	50.5%	(40.6%, 60.4%)	95.3%	(93.1%, 96.8%)	48	47	484	24	50.0%	51.0%	51.9%	96.0%
50%	49.5%	(39.6%, 59.4%)	95.9%	(93.8%, 97.3%)	47	48	487	21	50.0%	49.0%	50.6%	96.6%
45%	42.1%	(32.7%, 52.2%)	96.5%	(94.5%, 97.7%)	40	55	490	18	41.3%	42.9%	45.7%	96.6%
40%	35.8%	(26.9%, 45.8%)	97.8%	(96.2%, 98.8%)	34	61	497	11	34.8%	36.7%	38.3%	97.9%
35%	30.5%	(22.2%, 40.4%)	98.2%	(96.7%, 99.1%)	29	66	499	9	28.3%	32.7%	32.1%	98.3%
30%	25.3%	(17.6%, 34.8%)	98.8%	(97.4%, 99.5%)	24	71	502	6	26.1%	24.5%	25.9%	98.7%
25%	20.0%	(13.2%, 29.1%)	99.0%	(97.7%, 99.6%)	19	76	503	5	19.6%	20.4%	19.8%	98.9%
20%	17.9%	(11.5%, 26.8%)	99.6%	(98.6%, 99.9%)	17	78	506	2	19.6%	16.3%	17.3%	99.6%
15%	13.7%	(8.2%, 22%)	99.8%	(98.9%, 100%)	13	82	507	1	15.2%	12.2%	14.8%	99.8%
10%	8.4%	(4.3%, 15.7%)	100.0%	(99.2%, 100%)	8	87	508	0	8.7%	8.2%	8.6%	100.0%
5%	5.3%	(2.3%, 11.7%)	100.0%	(99.2%, 100%)	5	90	508	0	6.5%	4.1%	6.2%	100.0%
0%	0.0%	(0%, 3.9%)	100.0%	(99.2%, 100%)	0	95	508	0	0.0%	0.0%	0.0%	100.0%

Supplementary Table 4. Median ADAE score and rate of positivity at prespecified 95% sensitivity threshold by histopathological diagnoses.

Histopathologic diagnosis	Count	Proportion above threshold	Median ADAE score (log)
**Melanoma**	**95**	**97%**	**-1.58**
**Invasive melanoma**	**46**	**96%**	**-1.69**
**Melanoma in situ**	**49**	**98%**	**-1.58**
**Melanoma (unequivocal)**	**81**	**96%**	**-1.47**
**Non-melanoma**	**508**	**63%**	**-6.07**
**Atypical melanocytic proliferation**	**28**	**89%**	**-4.51**
**Keratinocyte carcinoma**	**22**	**100%**	**-4.27**
Basal cell carcinoma	9	100%	-4.45
Squamous cell carcinoma in situ	13	100%	-4.19
**Nevus**	**312**	**45%**	**-7.20**
With atypia	254	45%	-7.22
High-grade	*69*	*48%*	*-7.04*
Low-grade	*185*	*44%*	*-7.34*
Nevus other	58	45%	-7.16
**Other**	**146**	**89%**	**-4.65**
Actinic Keratosis	13	92%	-4.20
Lentigo	47	87%	-5.07
Seborrheic keratosis	23	74%	-5.24
Collison lesion	32	94%	-4.42
**Non-melanoma (unequivocal)**	**476**	**61%**	**-6.17**

Supplementary Table 5. Distributions of enrolled and non-enrolled study lesions.

P-values derived from Wilcoxon rank sum tests and Pearson's Chi-squared test.

Characteristic		Enrolled biopsied lesions (N=603)		Non-enrolled biopsied lesions (N=408)		p-value
		N (Col %)	Median (IQR)	N (Col %)	Median (IQR)	
ADAE score		-	-5.42 (-7.48, -3.60)	-	-5.94 (-7.32, -4.25)	0.082
Histopathologic diagnosis	Melanoma	95 (16%)	-	25 (6.1%)	-	<0.001
	Non-melanoma	508 (84%)	-	383 (94%)	-	
Study dermatologist	Derm1	389 (65%)	-	318 (78%)	-	<0.001
	Derm2	91 (15%)	-	24 (5.9%)	-	
	Derm3	56 (9.3%)	-	35 (8.6%)	-	
	Derm4	28 (4.6%)	-	6 (1.5%)	-	
	Derm5	22 (3.6%)	-	25 (6.1%)	-	
	Derm6-Derm11	17 (2.8%)	-	NA	-	
Age		-	61 (50, 72)	-	62 (54, 49)	0.148
Sex	Female	324 (54%)	-	248 (61%)	-	0.026
	Male	279 (46%)	-	160 (39%)	-	
Location	Head/neck	66 (11%)	-	76 (19%)	-	<0.001
	Torso	288 (48%)	-	139 (34%)	-	
	Upper extremity	117 (19%)	-	114 (28%)	-	
	Lower extremity	127 (21%)	-	76 (19%)	-	
	Palms and soles	5 (0.8%)	-	2 (0.5%)	-	
	Mucosal	0 (0%)	-	1 (0.2%)	-	

Supplementary Table 6. Dermatologist confidence before and after AI exposure.

Confidence scores ranged from 1 (lowest) to 4 (highest).

**Treating Dermatologist**	***Confidence***	**Frequency**		**Ordered Logistic Regression**			
		Pre-AI	***Post-AI***	***OR***	***95% CI***		***P-value***
Derm1	4	84%	68%	0.4258	0.301	0.598	<0.001
	3	8%	20%				
	2	7%	10%				
	1	1%	2%				
Derm2	4	18%	20%	2.1555	1.262	3.714	0.005
	3	27%	47%				
	2	31%	25%				
	1	24%	8%				
Derm3	4	38%	34%	0.8464	0.433	1.651	0.625
	3	21%	20%				
	2	21%	25%				
	1	20%	21%				
Derm4	4	25%	25%	0.6694	0.247	1.783	0.424
	3	54%	39%				
	2	21%	32%				
	1	0%	4%				
Derm5	4	9%	18%	1.8283	0.574	6.042	0.311
	3	27%	32%				
	2	64%	50%				
	1	0%	0%				

Supplementary Table 7. Calibration and discrimination measures of each study dermatologist.

P-values derived from one-sample t-tests, Kolmogorov-Smirnov (K-S) tests, and DeLong tests.

Treating Dermatologist (Cases contributed)	Melanoma Prevalence		Mean [Calibration]						Discrimination			
			Mean	*95% CI*	*Difference*	*P-value (t-test)*	*P-value (K-S)*	*Improvement*	*AUC*	*95% CI*	*Change*	*P-value (DeLong)*
Derm1 (n = 398)	5.9%	*Pre-AI probability*	15.0%	13.4%-16.6%	9.1%	<0.001	<0.001	-0.038	0.727	0.627-0.828	0.070	0.292
		Post-AI probability	18.8%	16.4%-21.2%	12.9%	<0.001			0.797	0.715-0.879		
Derm2 (n = 91)	28.6%	*Pre-AI probability*	21.4%	16.3%-26.6%	-7.2%	0.007	0.873	0.012	0.756	0.644-0.867	0.039	0.349
		Post-AI probability	22.6%	16.7%-28.5%	-6.0%	0.045			0.795	0.677-0.913		
Derm3 (n = 56)	51.8%	*Pre-AI probability*	47.1%	37.2%-57.0%	-4.7%	0.341	1.000	0.018	0.695	0.556-0.835	0.040	0.017
		Post-AI probability	48.9%	38.7%-59.1%	-2.9%	0.573			0.735	0.603-0.867		
Derm4 (n = 28)	0.0%	*Pre-AI probability*	8.8%	5.5%-12.1%	8.8%	<0.001	0.541	-0.066	*N/A*			
		Post-AI probability	15.4%	8.2%-22.6%	15.4%	<0.001						
Derm5 (n = 22)	50.0%	*Pre-AI probability*	28.2%	17.4%-38.9%	-21.8%	<0.001	0.860	0.070	0.793	0.600-0.987	0.116	0.105
		Post-AI probability	35.2%	22.7%-47.7%	-14.8%	0.023			0.909	0.791-1.000		

Supplementary Table 8. Dermatologist pre-AI and post-AI predicted melanoma probability, stratified by histopathological diagnosis.

Diagnosis	Count	Median pre-AI predicted probability	Median post-AI predicted probability	Post-AI biopsy decision (%)
**Melanoma**	**95**	**40**	**50**	**96%**
Unequivocal	*81*	*50*	*60*	*95%*
**Non-melanoma**	**508**	**10**	**5**	**66%**
**Atypical melanocytic proliferation**	**28**	**10**	**22.5**	**82%**
**Keratinocyte carcinoma**	**22**	**10**	**15**	**100%**
**Nevus**	**312**	**10**	**3**	**52%**
With atypia	254	10	3	52%
High-grade	*69*	*10*	*5*	*57%*
Low-grade	*185*	*10*	*3*	*50%*
Nevus other	58	7.5	2	50%
**Other**	**146**	**10**	**25**	**90%**
Actinic Keratosis	13	10	25	92%
Lentigo	47	10	30	87%
Seborrheic keratosis	23	5	20	83%
Collison lesion	32	10	25	91%

Supplementary Figure 1. Effect of dermoscopy image type on ADAE output and discrimination. (A) distributions of ADAE score in melanoma and non-melanoma, stratified by dermoscopy image type. The center line within each boxplot represents the median. The lower and upper hinges of the box represent the first and third quartiles (Q1 and Q3). The upper end of each whisker represents the more extreme value between the largest observed value and Q3 + 1.5 * IQR and the lower end of each whisker represents the more extreme value between the smallest observed value and Q1 - 1.5 * IQR, where IQR is the interquartile range. (B) ROC curves, stratified by dermoscopy image type. The purple line represents the predetermined threshold.

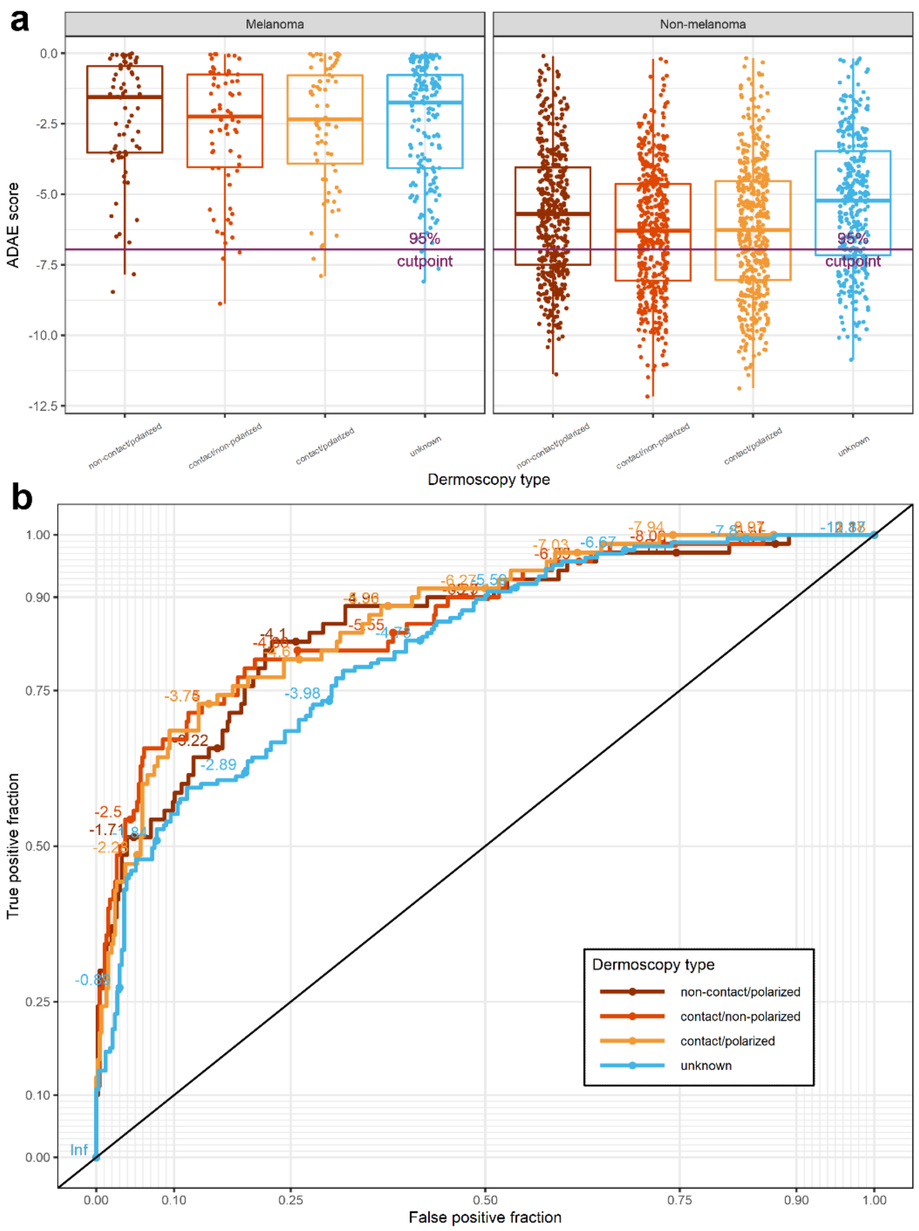

Supplementary Figure 2. ROC curves for images selected by study dermatologists vs. random non-selected image.

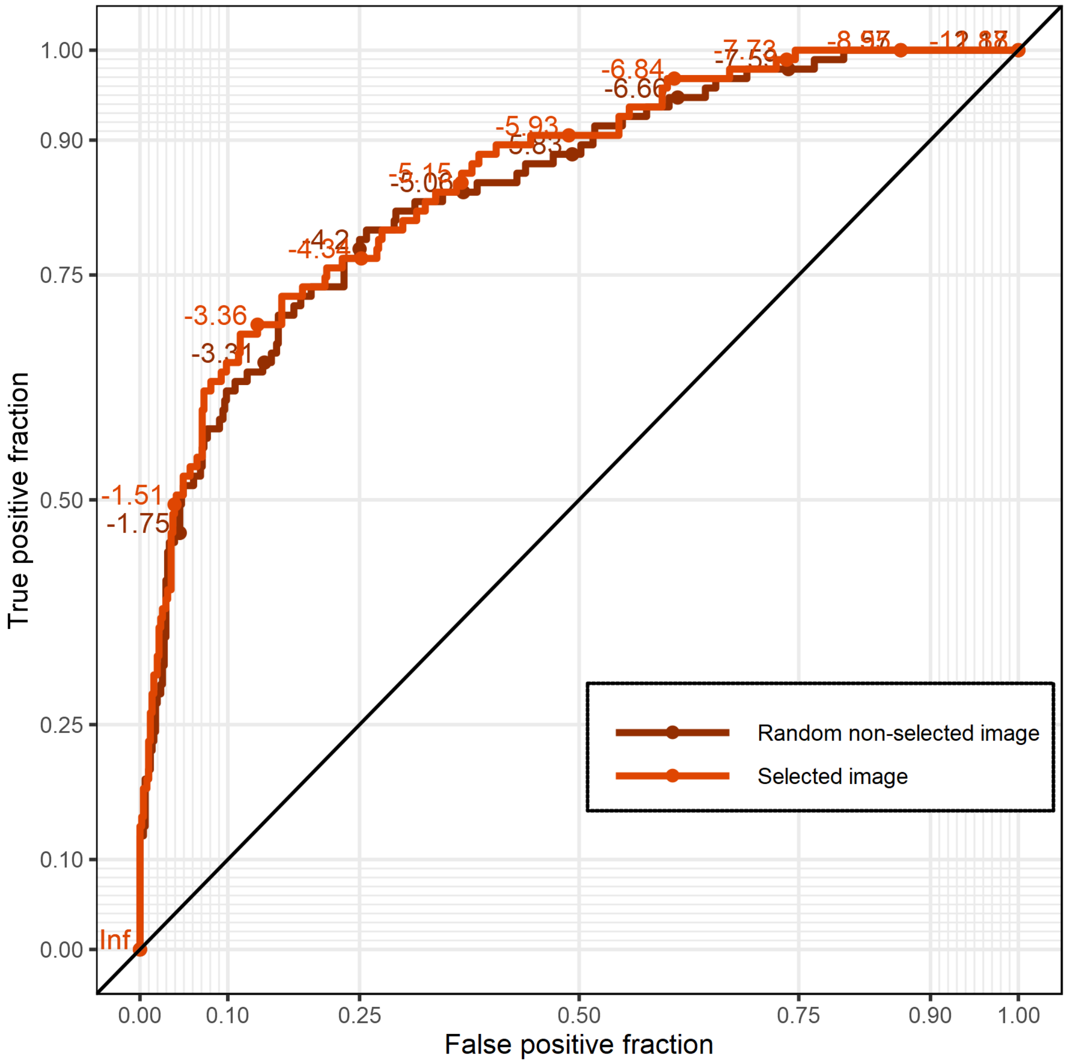

Supplementary Figure 3. ADAE performance on enrolled vs. non-enrolled biopsied lesions. (A) ROC for enrolled versus non-enrolled biopsied lesions. (B) Concordance of expected sensitivity with observed accuracy for enrolled versus non-enrolled biopsied lesions.

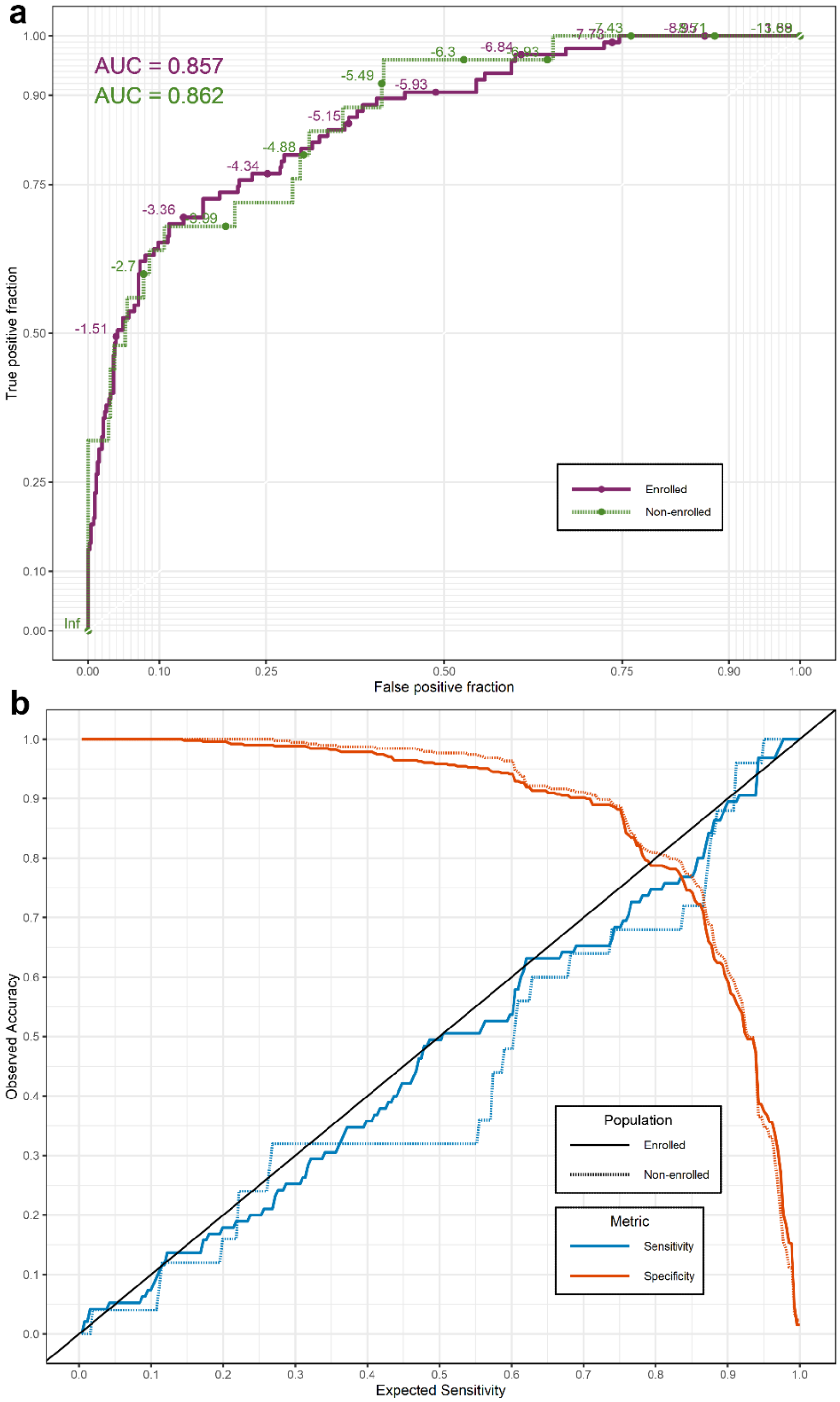

Supplementary Figure 4. ADAE scores for enrolled and non-enrolled lesions, stratified by diagnosis and dermatologist. The center line within each boxplot represents the median. The lower and upper hinges of the box represent the first and third quartiles (Q1 and Q3). The upper end of each whisker represents the more extreme value between the largest observed value and Q3 + 1.5 * IQR and the lower end of each whisker represents the more extreme value between the smallest observed value and Q1 - 1.5 * IQR, where IQR is the interquartile range. (B) ROC curves, stratified by dermoscopy image type. The purple line represents the predetermined threshold.

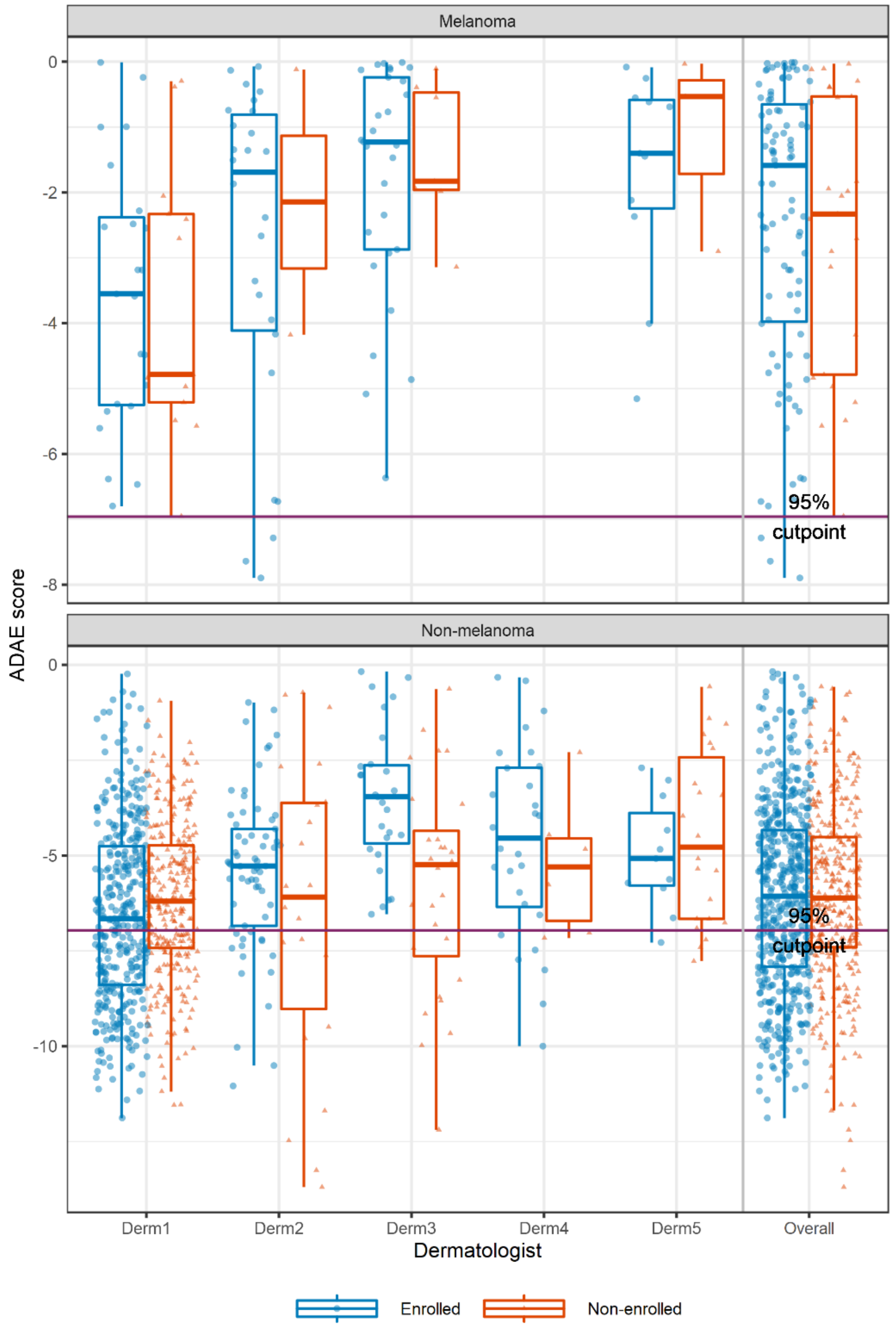

Supplementary Figure 5. Effect of ADAE exposure on dermatologist predicted melanoma probabilities. (A). Dermatologist predicted probability of melanoma for each study lesion (yellow dots – melanoma; blue dots – non-melanoma) before exposure to AI (x-axis) and after exposure to AI (y-axis). Dots on the solid black line signify lesions without any change in the predicted probability after AI exposure. Dots below the black line indicate that exposure to AI decreased the predicted melanoma probability. Dots above the black line indicate that exposure to AI increased the predicted melanoma probability. LOESS transform used to produce trend lines. (solid yellow line – melanoma; solid blue line – non-melanoma) with 95% confidence intervals (gray shading). (B) Dermatologist predicted probability of melanoma for each study lesion (yellow dots – melanoma; blue dots – non-melanoma) before exposure to AI (x-axis) and after exposure to AI (y-axis), stratified by individual study dermatologist. Pre-AI: before exposure to AI results; Post-AI: after exposure to AI results.

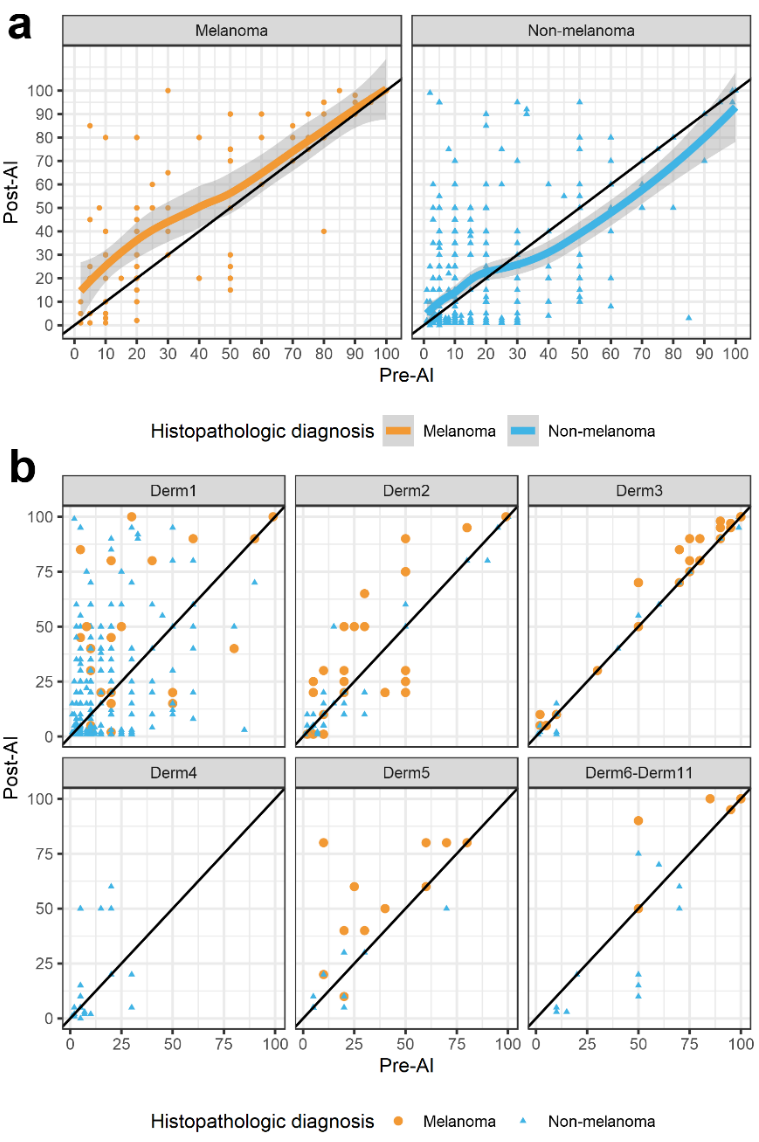

Supplement: Supplementary file 1 — Supplementary Materials [file 41746_2023_872_MOESM1_ESM.pdf]
