# Supplementary material for: Prospective validation of dermoscopy-based open-source artificial intelligence for melanoma diagnosis (PROVE-AI study)
Source: NPJ Digit Med. 2023 Jul 12;6:127. doi: 10.1038/s41746-023-00872-1 (PMC10338483; doi:10.1038/s41746-023-00872-1)
Supplement: Supplementary file 2 — Reporting Summary [file 41746_2023_872_MOESM2_ESM.pdf]

nature portfolio

Corresponding author(s): Veronica Rotemberg

Last updated by author(s): 03-10-2023

Reporting Summary

Nature Portfolio wishes to improve the reproducibility of the work that we publish. This form provides structure for consistency and transparency in reporting. For further information on Nature Portfolio policies, see our Editorial Policies and the Editorial Policy Checklist.

Statistics

For all statistical analyses, confirm that the following items are present in the figure legend, table legend, main text, or Methods section.

n/a	Confirmed	
☐	☒	The exact sample size (n) for each experimental group/condition, given as a discrete number and unit of measurement
☐	☒	A statement on whether measurements were taken from distinct samples or whether the same sample was measured repeatedly
☐	☒	The statistical test(s) used AND whether they are one- or two-sided<br>*Only common tests should be described solely by name; describe more complex techniques in the Methods section.*
☐	☒	A description of all covariates tested
☐	☒	A description of any assumptions or corrections, such as tests of normality and adjustment for multiple comparisons
☐	☒	A full description of the statistical parameters including central tendency (e.g. means) or other basic estimates (e.g. regression coefficient) AND variation (e.g. standard deviation) or associated estimates of uncertainty (e.g. confidence intervals)
☐	☒	For null hypothesis testing, the test statistic (e.g. F, t, r) with confidence intervals, effect sizes, degrees of freedom and P value noted<br>*Give P values as exact values whenever suitable.*
☒	☐	For Bayesian analysis, information on the choice of priors and Markov chain Monte Carlo settings
☒	☐	For hierarchical and complex designs, identification of the appropriate level for tests and full reporting of outcomes
☒	☐	Estimates of effect sizes (e.g. Cohen's d, Pearson's r), indicating how they were calculated

Our web collection on statistics for biologists contains articles on many of the points above.

Software and code

Policy information about availability of computer code

Data collection	Images collected as part of this study have been made available for public noncommercial use at this location: https://api.isic-archive.com/collections/170/
Data analysis	The ADAE algorithm is available via the kaggle site here: https://www.kaggle.com/competitions/siim-isic-melanoma-classification/discussion/175412 and can be used to perform the analyses and the specific ensembling we used will be made on github upon publication (and is exhaustively described in the supplement).

For manuscripts utilizing custom algorithms or software that are central to the research but not yet described in published literature, software must be made available to editors and reviewers. We strongly encourage code deposition in a community repository (e.g. GitHub). See the Nature Portfolio guidelines for submitting code & software for further information.

Data

Policy information about availability of data

All manuscripts must include a data availability statement. This statement should provide the following information, where applicable:

- Accession codes, unique identifiers, or web links for publicly available datasets
- A description of any restrictions on data availability
- For clinical datasets or third party data, please ensure that the statement adheres to our policy

Data sharing: All dermoscopy images analyzed for the patients who consented for the prospective study are freely available through the International Skin Imaging

Collaboration's online image archive at https://isic-archive.com. The ADAE code is also open source and hosted both on github and on kaggle, but please contact us with any questions or additional information requests.

Human research participants

Policy information about studies involving human research participants and Sex and Gender in Research.

Reporting on sex and gender	We have used patient and participant-reported sex throughout this paper.
Population characteristics	We have reported the population characteristics and covariates tested in Table 1
Recruitment	Patients who had consented for a skin biopsy of a lesion to exclude cutaneous melanoma (as determined by their dermatologist) were eligible. Participants ≥18 years of age were enrolled from dermatology clinics in New York and New Jersey from September 30, 2021, to June 24, 2022. Previously biopsied, recurrent, and mucosal lesions, as well as lesions removed for cosmetic purposes, were not eligible. Reporting of data followed the STARD checklist.
Ethics oversight	Memorial Sloan Kettering Cancer Center IRB

Note that full information on the approval of the study protocol must also be provided in the manuscript.

Field-specific reporting

Please select the one below that is the best fit for your research. If you are not sure, read the appropriate sections before making your selection.

☒ Life sciences ☐ Behavioural & social sciences ☐ Ecological, evolutionary & environmental sciences

For a reference copy of the document with all sections, see nature.com/documents/nr-reporting-summary-flat.pdf

Life sciences study design

All studies must disclose on these points even when the disclosure is negative.

Sample size	The primary aim was to assess the reliability of ADAE's sensitivity for melanoma classification on prospectively acquired cases at a predefined threshold corresponding to 95% sensitivity on a multi-institutional dataset curated for the 2020 SIIM-ISIC Melanoma Classification challenge. We defined a non-inferiority test with an acceptable margin no greater than 5%. Through a Monte Carlo random sampling approach, it was determined that 86 melanoma cases would power the study at 80% to demonstrate a true-positive fraction greater than 90% if the parametric sensitivity is indeed 95%. Eighty-six melanomas were estimated to be a reasonable target across 9-months of accrual based on the frequency of melanoma diagnosed at the institution in recent years.
Data exclusions	We excluded biopsies performed for cosmetic purposes, previously biopsied lesions, and those on mucosal surfaces as they are inconvenient to image or where AI might not be of assistance to the treating clinician.
Replication	n/a
Randomization	n/a
Blinding	Investigators were not blinded

Reporting for specific materials, systems and methods

We require information from authors about some types of materials, experimental systems and methods used in many studies. Here, indicate whether each material, system or method listed is relevant to your study. If you are not sure if a list item applies to your research, read the appropriate section before selecting a response.

Materials & experimental systems

n/a	Involved in the study	
☒	☐	Antibodies
☒	☐	Eukaryotic cell lines
☒	☐	Palaeontology and archaeology
☒	☐	Animals and other organisms
☐	☒	Clinical data
☒	☐	Dual use research of concern

Methods

n/a	Involved in the study	
☒	☐	ChIP-seq
☒	☐	Flow cytometry
☒	☐	MRI-based neuroimaging

Clinical data

Policy information about clinical studies

All manuscripts should comply with the ICMJE guidelines for publication of clinical research and a completed CONSORT checklist must be included with all submissions.

Clinical trial registration

NCT04743362

Study protocol

Protocol is available upon request - but is a substudy of a larger protocol so cannot be made public

Data collection

September 30,2021-June 24, 2022

Outcomes

Primary outcomes:
Sensitivity: True positive / (True positive + False Negative)
Specificity: True negative / (True negative + False Positive)
Net benefit: (True positives / Population) – (False Positives / Population) X (risk threshold / (1-risk threshold))
Net avoidable biopsies: (True negatives / Population) – (False Negatives / Population) / (risk threshold / (1-risk threshold))
Number needed to biopsy: (True positives + false positives) / true positives
